# Sports Nutrition Ingredients and Governance, Exercise Training, and Sports Technology

**DOI:** 10.1007/s40279-023-01960-8

**Published:** 2023-11-21

**Authors:** Lawrence L. Spriet

**Affiliations:** Human Health and Nutritional Sciences, University of Guelph, Guelph, ON N1G 2W1 Canada

This Sports Medicine supplement aims to provide recent information in a variety of fields with the common goal of optimising athlete performance and health. In the constantly evolving field of sports nutrition, for example, the use of carbohydrates to improve soccer skill is examined and the importance of vitamins and minerals for athletic performance is reviewed, including when supplements may be needed in athletic populations. This supplement also reviews the latest information on the use of buffering agents and creatine supplements to improve sports performance at the levels of skeletal muscle and the brain, as well as approaches to avoid inadvertent anti-doping violations. Furthermore, the supplement includes a perspective on the value of high-intensity interval training (HIIT) for athletic performance and health. The supplement concludes by examining the use of technology to collect many forms of data from elite athletes while training and competing. It discusses the issues surrounding the use of technology to improve performance and the guardrails needed to protect the athletes who provide the data.

The Gatorade Sports Science Institute (GSSI) has been bringing sports nutrition and sports science researchers together for nearly four decades to discuss many topics that relate to the nutrition, performance, and well-being of athletes. Since 2012 these meetings have been known as the GSSI Expert Panel. The 2022 meeting was the first in-person event since the worldwide COVID-19 pandemic and was a great success with speakers from the USA, Canada, Australia, and the UK. Following the meeting, the authors summarised the recent work in their topic area, resulting in the manuscripts in this Sports Medicine supplement (the tenth in a series supported by GSSI).

The first paper in this supplement examines the potential importance of a carbohydrate intake for skill performance in soccer players [1]. A variety of sport-specific skills must be performed during a soccer match, often at very high running speeds, and the onset of mental and physical fatigue impairs skill performance. While it is clear that a carbohydrate-rich diet before a soccer match and ingesting carbohydrate during a match delays fatigue, carbohydrate also appears to play a role in maintaining skill level. Because of the dynamic nature of the game, research to examine the role of carbohydrate on soccer skill has been conducted in controlled non-game environments, where the role of competition has been ruled out. The applicability of this work to maintaining skill performance in soccer is discussed. The second paper in this supplement discusses the importance of several vitamins and minerals (iron, B vitamins, calcium, vitamin D, folate) that are essential for athletic success [2]. An issue is whether athletes who engage in heavy training ever experience the need to supplement with these crucial components of energy production and athletic performance. Normally, replenishment of vitamins and minerals would be accomplished through a diet that meets the energy needs of the athlete. If this does not occur, practitioners are encouraged to use a framework that assesses the energy requirements, dietary practices, and biological and clinical status of the athletes to decide whether supplementation is needed.

The next two papers examine the use of common supplements, or ergogenic aids, designed to improve athletic performance [3, 4]. The supplemental use of buffers (sodium bicarbonate, sodium citrate and beta-alanine) is examined in three contexts: highly trained female athletes, extreme environments including altitude and heat camps, and when buffers are combined [3]. Surprisingly, only ~ 20% of the studies examined reported a performance improvement as several limitations related to study designs, supplement dose and timing, exercise test duration and intensity, and recruitment of highly trained individuals (especially women) limited the evaluation. Future research should help clarify the efficacy of these buffers in different athletic populations and environments. Creatine supplementation has been available since 1992 and has focussed on the ability of this supplement to augment the total creatine and phosphocreatine stores in skeletal muscle, setting up the possibility of improving performance in short-term high intensity efforts. Creatine supplementation also appears to augment the anabolic effects of resistance training. However, the present paper goes further and examines the emerging role of creatine supplementation for brain health and function [4]. Importantly, creatine supplementation increases brain creatine stores and improves some aspects of cognition and memory, mainly in older adults or in younger adults when under metabolic stress such as sleep deprivation. It may also aid recovery from traumatic brain injury and reduce symptoms of depression and anxiety, although this work is preliminary.

The fifth paper in this supplement stresses the importance of a behaviourally informed approach to reduce the risk of inadvertent anti-doping violations from supplement use in athletes [5]. Supplement use is high in many athletic populations, and indiscriminate supplement use could also be a risk to athlete health. Many screening programmes have been established to minimise the chance of ingesting a prohibited substance, recognising that supplement use involves a myriad of behaviours. This review outlines a behaviourally informed approach to minimise supplement use risk and examines the existing research through the lens of the Behaviour Change Wheel.

High-intensity interval training has been used extensively to promote adaptations in a time-efficient manner in populations of all levels of fitness—from untrained individuals to highly trained athletes. Simply put, it is “repeated bouts of relatively hard work, interspersed with recovery periods of easier work or rest” [6]. This perspective paper suggests that the interpretation and application of interval training are hampered by the lack of standardised terminology. This is especially true when defining “high-intensity”. It is proposed that in a performance context, HIIT can be characterised as intermittent exercise bouts performed above the heavy intensity domain. This can be determined by measuring critical power/speed, second lactate threshold, maximal lactate steady state, or lactate turn point. In a health context, HIIT can be characterised as intermittent bouts of exercise performed above moderate intensity, which can be determined by measuring perceived exertion, oxygen uptake, or heart rate as defined in public health and exercise prescription guidelines. The review suggests that further research is needed to clarify responses to different HIIT strategies and offers a conceptual framework that outlines how HIIT can be defined and operationalised within both the performance and health contexts.

The final paper examines the growing number of wearable sensors and software technologies that are used to monitor, analyse, and transmit data from humans in real time. While the interest in the sporting world is high, these technologies can also be used in the biomedical and media industries. This paper describes the use of wearable technology in high profile sporting events where academic and industry partners worked together to implement real-time wearable solutions to protect the health of athletes competing in hot and humid environments. Despite the potential benefits of this technology, there are many concerns regarding its use, including the lack of peer-reviewed research examining the possible benefits of the data, the quality of hardware and provided data, information overload, data security, and exaggerated marketing claims. Athletes require protection and the present need is to overcome ethical/data concerns and develop wearable technologies that are backed by quality science. The fields of sport/exercise science and medicine provide a platform to understand the impact of wearable sensors on performance and health.

This supplement includes papers examining several aspects of sports nutrition ingredients and supplements, athlete governance, exercise training, and wearable sport technology that may positively impact the mental and physical well being, health, and performance of athletes. These reviews provide excellent insight into where new research needs to move. It is hoped that these papers will encourage the readers to disseminate the existing knowledge related to these topics and generate new knowledge to help answer the many remaining questions.
